# Comparative studies of TIMP-1 immunohistochemistry, TIMP-1 FISH analysis and plasma TIMP-1 in glioblastoma patients

**DOI:** 10.1007/s11060-016-2252-4

**Published:** 2016-09-12

**Authors:** Charlotte Aaberg-Jessen, Bo Halle, Stine S. Jensen, Sven Müller, Unni Maria Rømer, Christian B. Pedersen, Nils Brünner, Bjarne W. Kristensen

**Affiliations:** 1 Department of Pathology, Odense University Hospital, Winsløwparken 15, 3. Floor, 5000 Odense, Denmark; 2 Department of Nuclear Medicine, Odense University Hospital, Odense, Denmark; 3 Department of Neurosurgery, Odense University Hospital, Odense, Denmark; 4 Section of Molecular Disease Biology, Department of Veterinary Disease Biology, Faculty of Health and Medical Sciences, University of Copenhagen, Copenhagen, Denmark; 5 DAKO, Glostrup, Denmark; 6 Institute of Clinical Research, University of Southern Denmark, Odense, Denmark

**Keywords:** Glioblastoma, Glioma, TIMP-1, Fluorescence in situ hybridization, Plasma, Immunohistochemistry

## Abstract

Tissue inhibitor of metalloproteinases-1 (TIMP-1) has been associated with poor prognosis and resistance towards chemotherapy in several cancer forms. In a previous study we found an association between a low TIMP-1 tumor immunoreactivity and increased survival for glioblastoma patients, when compared to moderate and high TIMP-1 tumor immunoreactivity. The aim of the present study was to further evaluate TIMP-1 as a biomarker in gliomas by studying *TIMP-1* gene copy numbers by fluorescence in situ hybridization (FISH) on 33 glioblastoma biopsies and by measuring levels of TIMP-1 in plasma obtained pre-operatively from 43 patients (31 gliomas including 21 glioblastomas) by enzyme-linked immunosorbent assay (ELISA). The results showed *TIMP-1* gene copy numbers per cell ranging from 1 to 5 and the *TIMP-1*/CEN-X ratio ranging between 0.7 and 1.09, suggesting neither amplification nor loss of the *TIMP-1* gene. The TIMP-1 protein levels measured in plasma were not significantly higher than TIMP-1 levels measured in healthy subjects. No correlation was identified between TIMP-1 tumor cell immunoreactivities and the *TIMP-1* gene copy numbers or the plasma TIMP-1 levels. In conclusion, high immunohistochemical TIMP-1 protein levels in glioblastomas were not caused by TIMP-1 gene amplification and TIMP-1 in plasma was low and not directly related to tumor TIMP-1 immunoreactivity. The study suggests that TIMP-1 immunohistochemistry is the method of choice for future clinical studies evaluating TIMP-1 as a biomarker in glioblastomas.

**Electronic supplementary material:**

The online version of this article (doi:10.1007/s11060-016-2252-4) contains supplementary material, which is available to authorized users.

## Introduction

The current standard treatment of the highly malignant and most frequent brain tumor, the glioblastoma, includes surgery, radiotherapy and chemotherapy. However, glioblastomas remain highly treatment-resistant and thereby incurable [1]. In the last decade, the median overall survival has increased to 14.6 months and the 2-year survival has increased from 10.9 to 27.2 %, mainly due to the introduction of temozolomide [2]. This improved survival has increased the interest in prognostic and predictive biomarkers for this group of patients.

In a previous study, we characterized the protein expression of the tissue inhibitor of metalloproteinases-1 (TIMP-1) in astrocytic brain tumors and found that the protein expression increased with tumor grade (World Health Organisation (WHO) grade II–IV). Moreover we found that glioblastoma patients with a low TIMP-1 expression had a significant longer overall survival than patients with a moderate or high TIMP-1 protein expression [3]. In line with our report, high TIMP-1 protein expression levels in tumor tissue or in plasma obtained pre-operatively have been associated with poor prognosis in breast and colorectal cancer amongst others [4–12].

Immunohistochemistry can be difficult to quantify and can be dependent on pre-analytical factors such as hypoxia, time and type of fixation as well as analytical variables such as choice of antibody and type of antigen retrieval method. In addition to this, gliomas can be difficult to reach surgically and are known for being highly necrotic. The necrosis may comprise up to 80 % of the tumor area and is a diagnostic hallmark in glioblastoma [1]. Therefore, vital tumor tissue is often sparse in contrast to blood samples, which are easier to obtain. In this study, we aimed to determine (1) whether assessments of *TIMP-1* gene copy number in the tumor cells and/or (2) measurement of plasma TIMP-1 protein levels could substitute TIMP-1 immunohistochemistry in glioma research.

In order to investigate the *TIMP-1* gene copy number, we produced a new TIMP-1 probe suitable for fluorescence in situ hybridization (FISH) and estimated the *TIMP-1*/Centromere region on chromosome X (CEN-X) ratio in 33 glioblastoma biopsies. The ratios were correlated to the TIMP-1 immunoreactivity in the same biopsies. In addition, blood samples and biopsies were collected from 43 brain tumor patients, comprising 20 glioblastoma patients, and the TIMP-1 plasma levels were correlated to TIMP-1 immunoreactivity in the matched biopsies.

The results showed neither amplification nor loss of the *TIMP-1* gene. The TIMP-1 levels measured in plasma were not significantly higher than the TIMP-1 levels measured in healthy matched controls. No significant correlations were identified between the immunohistochemical tumor cell TIMP-1 levels and the FISH results or the plasma TIMP-1 levels. The study thus suggests that TIMP-1 immunohistochemistry is the method of choice when studying TIMP-1 as a biomarker in glioblastomas.

## Materials and methods

### Patients included in the study

#### Cohort I

Blood samples and corresponding tumor tissue biopsies were collected from 43 patients who underwent initial surgery of a brain tumor at Odense University Hospital, Denmark between September 2009 and February 2010. All patients included had primary lesions not previously treated except the patient with a recurrent anaplastic oligoastrocytoma. Informed consent was obtained from patients beforehand and blood samples were collected at the initial visit prior to surgery. Control blood samples were collected from healthy donors after informed consent. Fresh tumor tissue biopsies from all tumor patients were fixed in 4 % neutral buffered formalin and paraffin embedded. Three µm sections were stained with haematoxylin eosin to define representative tumour regions. All samples were classified according to WHO guidelines 2007 [1].

#### Cohort II

Two tissue micro arrays (TMAs), consisting of 9 and 24 glioblastoma biopsies, respectively, were produced from archival material at the Department of Pathology, Odense University Hospital, Denmark, between 2004 and 2008.

The present study was approved by The Regional Scientific Ethical Committee (Approval Number S-20080086).

### FISH analysis

**Fig. 1:**
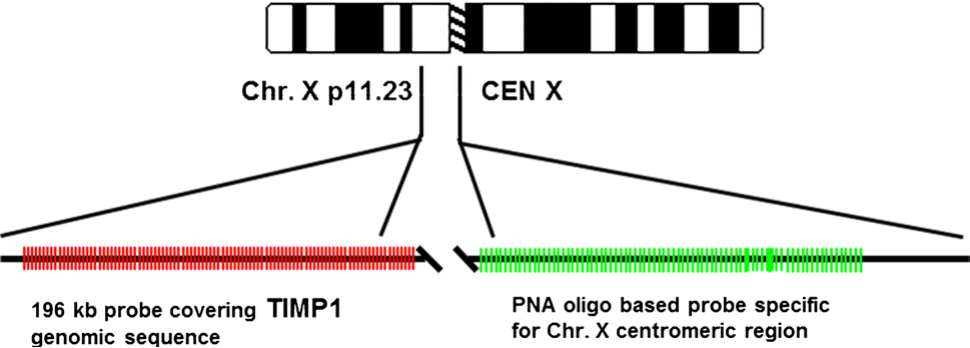
Schematic illustration of the *TIMP-1*/CEN-X probe mix localization on chromosome X. The *TIMP-1* targeting part of the probe consisted of Texas Red labeled BAC clone RP11-466C12. The BAC clone covers the entire *TIMP-1* genomic sequence and flanking regions. Start and end pos. according to UCSC Genome Browser Feb. 2009: 47392808–47588710. The chromosome X centromere targeting part of the probe consisted of a mixture of three FITC labeled Peptide Nucleic Acid oligos specific for the CEN-X α-satellite region

FISH analysis was performed on the two TMAs described above, in order to elucidate *TIMP-1* gene copy number. The TIMP-1 probe mixture was developed by Dako A/S. A schematic illustration of the *TIMP-1*/CEN-X probe mix localization on chromosome X is illustrated in Fig. 1. FISH was performed according to Dako Histology FISH Accessory Kit (K5599). In brief, paraffin sections were deparaffinized and rehydrated. Sections were placed in pre-treatment buffer and pre-treatment was performed using a microwave oven. Subsequently, the sections were incubated with RTU-pepsin for 2 min at 37 °C and washed twice for 3 min. Slides were air-dried for 15–20 min, followed by application of 10 µl probe mix. Hybridization was carried out for approximately 20 h using a melting temperature of 82 °C and a hybridization temperature of 45 °C. Coverslips were mounted using fluorescence mounting medium with DAPI counter staining of the cell nuclei. Detailed information on development of TIMP-1 probe mixture and FISH analysis is given in Online Resource 1.

### Assessment of *TIMP-1* copy numbers and *TIMP-1*/CEN-X ratios

Hybridization signals in the tumor cells in sections obtained from cohort II were scored using a Leica DM6000 microscope (Leica) equipped with a 100× oil-immersion objective (numeric aperture, 0.17). A dual-bandpass fluorescence filter (Leica) was used to visualize the fluorescein isothiocyanate (FITC) and Texas Red signals simultaneously. Sixty non-overlapping interphase nuclei with intact morphology, based on 4′,6-diamidino-2-phenylindole (DAPI) counterstaining, were scored to determine the number of hybridization signals for each *TIMP-1* gene (red color) and CEN-X (green color) and the *TIMP-1*/CEN-X ratio was estimated.

### Collection of blood samples and plasma TIMP-1 measurements

From cohort I, venous blood samples were collected in EDTA coated tubes (Becton-Dickenson) at the Department of Neurosurgery, Odense University Hospital, Denmark and placed on ice immediately. Within 1 h the plasma was separated from blood cells by centrifugation at 2500 RPM for 25 min. Subsequently, plasma was stored at −80 °C. A well-established sensitive and specific sandwich ELISA was used for measuring the total amount (complexed and non-complexed) TIMP-1 protein level in the plasma samples as described previously [13]. Blood samples from healthy donors were collected and processed in the same way.

### TIMP-1 immunohistochemistry

The TIMP-1 immunoreactivity was assessed on paraffin sections from both cohort I and cohort II. For cohort II formalin fixed paraffin embedded tissue (FFPE) TMA sections adjacent to the sections used for *TIMP-1* FISH were stained. In cohort I, TIMP-1 immunoreactivity was studied in whole mount sections from FFPE tissue. The TIMP-1 immunohistochemistry was performed using the monoclonal VT7 antibody [14] as described earlier [3]. Assessment of the immunohistochemical TIMP-1 expression was based on a semiquantitative microscopy-based scoring system used previously [3] evaluating the average percentages of TIMP-1 positive tumor cells and blood vessels and their average staining intensities, whereas necrotic areas and invasion zones were excluded. Regarding the percentage of positive tumor cells, the score 0 corresponds to 0 % to <2 % positive cells, score 1 to 2 % to <15 % positive cells, score 2 to 15 % to <40 % positive cells and score 3 to 40 % to 100 % positive cells. Regarding the tumor cell staining intensity, the score 0 corresponds to no staining, score 1 to faint staining, score 2 to moderate staining and score 3 to intense immunostaining. The percentage of positive tumor blood vessels and the blood vessel staining intensity were assessed in the same way in the biopsies, resulting in a total score between 0 and 12 for each biopsy. For comparison with the tumor cell *TIMP-1* gene copy numbers and *TIMP-1*/CEN-X ratios, the TIMP-1 immunoreactivity in the TMAs was only assessed in the tumor cells.

### Statistics

Estimation of Spearman’s rank correlation coefficient was performed between *TIMP-1*/CEN-X ratios and immunohistochemistry (IHC) scores as well as between plasma values and IHC scores using Graph Pad Prism 5.0 (Graphpad Software). For each patient the plasma TIMP-1 was compared to plasma TIMP-1 measured in two healthy subjects using a linear model with the TIMP-1 log transformed and adjusted for gender and age. The healthy subject samples are described in Nielsen et al. [7] and the analysis was performed using SAS (SAS v9.1, SAS Institute). The reporting recommendations for tumor marker prognostic studies (REMARK) guidelines [15] were followed wherever applicable.

## Results

### *TIMP-1* gene copy number and TIMP-1 immunoreactivity in glioblastoma

**Fig. 2:**
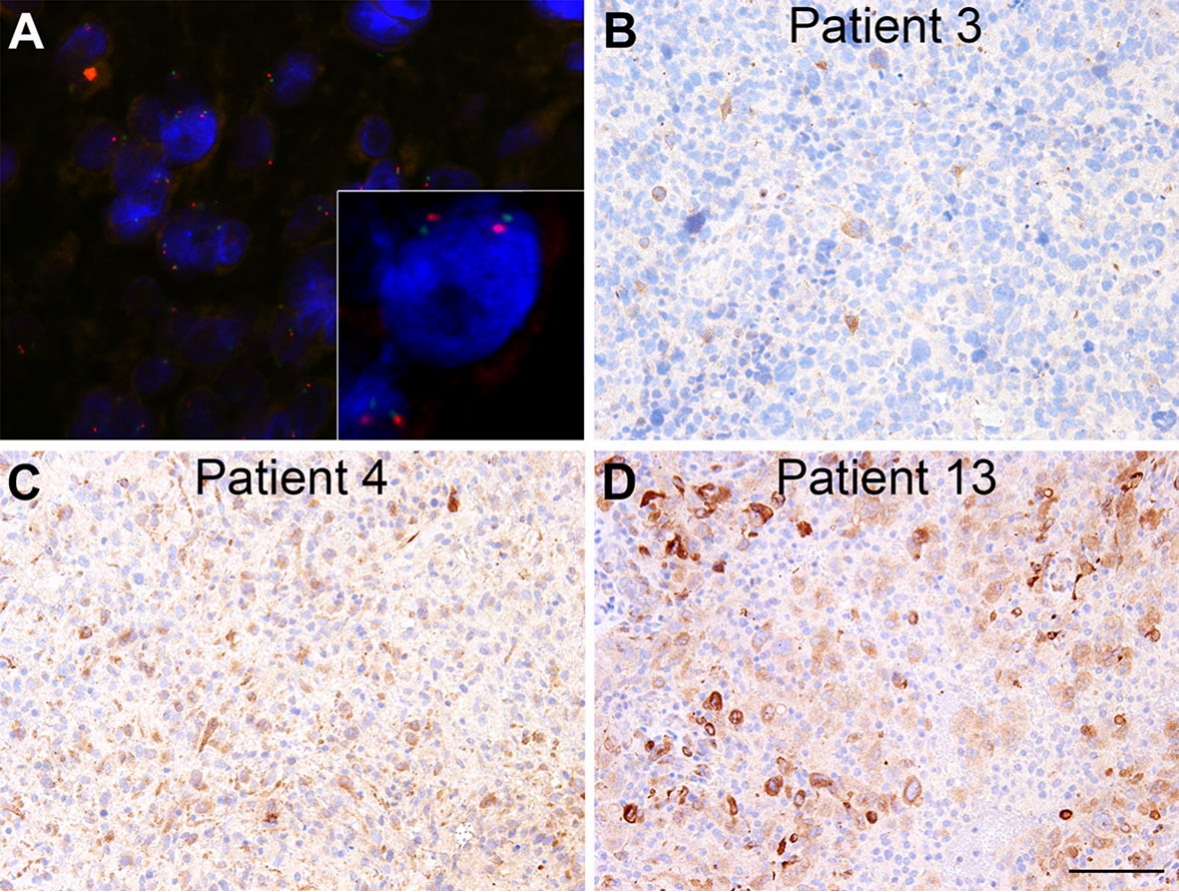
The ratio between *TIMP-1* genes (**a**, *red* signal) and CEN-X (**a**, *green* signal) was assessed using FISH in 33 glioblastoma biopsies. TIMP-1 immunohistochemistry was performed on adjacent paraffin sections (**b**–**d**) revealing a higher degree of variation of the TIMP-1 immunoreactivity. In glioblastomas with a FISH score of approximately 1, biopsies with a low TIMP-1 expression (**b**) as well as biopsies with a high TIMP-1 expression (**d**) were observed. A high TIMP-1 protein expression was found in the biopsy from patient 4 (**c**), whereas the TIMP-1 FISH analysis showed a low *TIMP-1*/CEN-X ratio of 0.7 (Table 1). *Scale bar* 100 µm

In cohort II, the *TIMP-1* gene, localized on chromosome X, was in general detected in one or two copies per nucleus depending on the gender of the patient. The ratio of *TIMP-1* genes (Fig. 2a, red signal) compared to the centromere signals (Fig. 2a, green signal) per nucleus was in general close to 1, but varied between 0.7 and 1.09, suggesting neither amplification nor loss of the *TIMP-1* gene (Table 1).

**Table 1:** TIMP-1 FISH analysis and TIMP-1 immunohistochemistry performed on TMAs consisting of 33 glioblastoma biopsies

Patient no.	Gender male/female	T1MP-1	CEN-X	Ratio	Total TIMP-1 IHC score (0–6)
1	M	71	71	1.00	3
2	M	76	78	0.97	4
3	F	201	185	1.09	4
4	F	96	137	0.70	5
5	M	60	60	1.00	2
6	M	112	108	1.04	2
7	M	65	65	1.00	2
8	F	109	108	1.01	2
9	M	61	62	0.98	3
10	F	110	110	1.00	5
11	M	60	60	1.00	6
12	F	173	167	1.04	3
13	M	64	61	1.05	6
14	M	61	60	1.02	3
15	M	75	73	1.03	4
16	M	122	114	1.07	4
17	M	70	68	1.03	3
18	F	115	116	0.99	4
19	F	104	103	1.01	3
20	M	68	68	1.00	5
21	F	108	103	1.05	4
22	M	60	60	1.00	3
23	M	139	139	1.00	3
24	F	134	137	0.98	2
25	M	60	60	1.00	4
26	M	122	120	1.02	0
27	M	61	61	1.00	0
28	M	80	77	1.04	2
29	M	60	60	1.00	5
30	M	65	66	0.98	2
31	F	121	121	1.00	3
32	M	74	79	0.94	3
33	F	99	97	1.02	4

The *TIMP-1* signals and CEN-X signals were counted in 60 cells per biopsy and the *TIMP-1*/CEN-X ratio was calculated. The TIMP-1 immunostaining was scored according to TIMP-1 positive tumor cells (0–3) and tumor cell staining intensity (0–3) resulting in a total IHC score between 0 and 6.

TIMP-1 immunohistochemistry, performed on adjacent paraffin sections from patients belonging to cohort II, revealed a high degree of variation of the TIMP-1 protein expression (Fig. 2b–d; Table 1). In glioblastomas with a *TIMP-1*/CEN-X ratio of approximately 1, biopsies with a low TIMP-1 expression (Fig. 2b) as well as biopsies with a high TIMP-1 expression (Fig. 2d) were observed. A high TIMP-1 protein expression was found in the biopsy from patient 4 (Fig. 2c), whereas the *TIMP-1* FISH analysis showed a low *TIMP-1*/CEN-X ratio of 0.7 (Table 1) in this biopsy. The TIMP-1 immunoreactivity in the tumor cells was scored, resulting in a total score between 0 and 6 for each biopsy.

We did not find any significant correlation between *TIMP-1* gene copy number and TIMP-1 immunoreactivity for females (Pearson r = 0.16), males (Pearson r = 0.25) or for all the patients combined, when taking the expected number of chromosome X into account (Pearson r = 0.27). Moreover, no correlations between the *TIMP-1*/CEN-X ratio and the total immunohistochemical scores (Pearson r = 0.13) were found.

### TIMP-1 in blood samples versus TIMP-1 reactivity in biopsies

The 43 brain tumor patients constituting cohort I are described in Table 2 and included 29 glioma patients of whom 20 were glioblastoma patients. The median value of plasma TIMP-1 in the 43 samples was 78.3 ng/ml (range 58.8–131.9 ng/ml) compared to a median value for healthy subjects of 74.8 ng/ml (range 65.0–84.3 ng/ml) (Table 2). For the glioblastomas, the median plasma TIMP-1 was 80.1 ng/ml (range 59.5–131.9 ng/ml) and 76.4 ng/ml (range 70.0–84.3 ng/ml) for the matched healthy subjects. Thus, no significant differences were seen between plasma TIMP-1 levels measured in brain tumor patients compared to healthy subjects. However, a slightly elevated TIMP-1 level was detected in a few patients (patient 7, 21 and 23 in Table 2), but no obvious pattern was recognized.

**Table 2:** Blood samples were collected from 43 brain tumor patients including 20 glioblastoma patients

Patient no.	Gender male/female	Age	Diagnosis	Plasma TIMP-1	Plasma reference	Upper 95 percentile	Total TIMP-1 IHC score
1	M	64	GBM	88.8	75.9	109.4	7
2	M	38	R-AOA	71.0	65.0	93.7	6
3	M	56	GBM	71.2	72.4	104.3	9
4	F	65	DA	62.8	73.4	105.7	8
5	M	77	M-PAC	75.5	82.1	118.2	6
6	M	65	SS	74.5	76.4	110.1	3
7	F	64	GBM	107.4	73.0	105.1	Na
8	F	62	GBM	64.6	72.1	103.9	8
9	F	58	DA	58.9	70.4	101.4	Na
10	F	56	GBM	60.3	70.0	100.2	6
11	M	61	GBM	63.6	74.6	107.5	6
12	M	72	GBM	59.5	79.6	114.7	6
13	F	70	AOD	70.7	75.6	108.9	3
14	M	53	NM	66.9	71.1	102.4	Na
15	M	75	M-LAC	65.6	81.1	116.8	7
16	M	72	GBM	73.4	79.6	114.7	10
17	F	66	M-BAC	69.4	73.8	106.4	5
18	F	72	GBM	61.8	76.5	110.2	6
19	M	46	DA	68.2	68.2	98.3	4
20	M	66	GBM	97.7	76.8	110.7	7
21	M	67	GBM	131.9	77.3	111.4	7
22	M	52	M-MM	80.3	70.7	101.8	4
23	F	67	LF	127.2	74.3	107.0	Na
24	M	40	AA	53.9	65.8	94.8	6
25	F	77	GBM	99.3	78.8	113.6	7
26	F	68	AOA	80.8	74.7	107.5	3
27	M	68	GBM	65.3	77.8	112.0	8
28	M	59	GBM	81.4	73.7	106.2	9
29	M	62	GBM	71.0	75.0	108.1	6
30	M	64	M-LAC	76.6	75.9	109.4	7
31	F	55	AA	66.1	69.1	99.6	8
32	M	68	GBM	75.4	77.8	112.0	3
33	M	70	AA	88.4	75.6	108.9	8
34	M	81	GBM	82.8	84.0	121.1	11
35	M	82	GBM	87.8	84.3	121.8	7
36	M	79	M-LAC	116.1	83.0	119.6	5
37	M	60	GBM	76.6	74.1	106.8	11
38	M	74	AA	78.2	80.6	116.1	Na
39	F	69	LF	81.8	75.2	108.3	8
40	M	46	AF	89.1	68.2	98.3	5
41	F	66	GBM	81.5	73.8	106.4	9
42	F	50	M-LAC	68.7	67.1	96.7	Na
43	M	52	M-LAC	74.2	70.7	101.8	0

Plasma TIMP-1 was measured and compared to two healthy controls matched by age and gender. Only two glioblastoma patients and one lymphoma patient were slightly above the upper 95 percentile (Patient 7, 21 and 23). TIMP-1 immunohistochemistry was performed on the corresponding biopsies from 37 of the patients. The TIMP-1 expression was scored according to TIMP-1 positive tumor cells (0–3), tumor blood vessels (0–3) as well as the staining intensities (0–3) resulting in a total score between 0 and 12

The diagnoses were: *DA* diffuse astrocytoma, *AA* anaplastic astrocytoma, *AOD* anaplastic oligoastrocytoma, *AOD* anaplastic oligodendroglioma, *GBM* glioblastoma, *R-AOA* recurrent anaplastic oligoastrocytoma, *AE* anaplastic ependynoma, *SS* synovial sarcoma, *LF* lymphoma, *M-LAC* metastasis from lung adenocarcinoma, *M-MM* metastasis from malignant melanoma, *M-PAC* metastasis from prostate adenocarcinoma, *M-BAC* metastasis from breast adenocarcinoma, *NA* not available

**Fig. 3:**
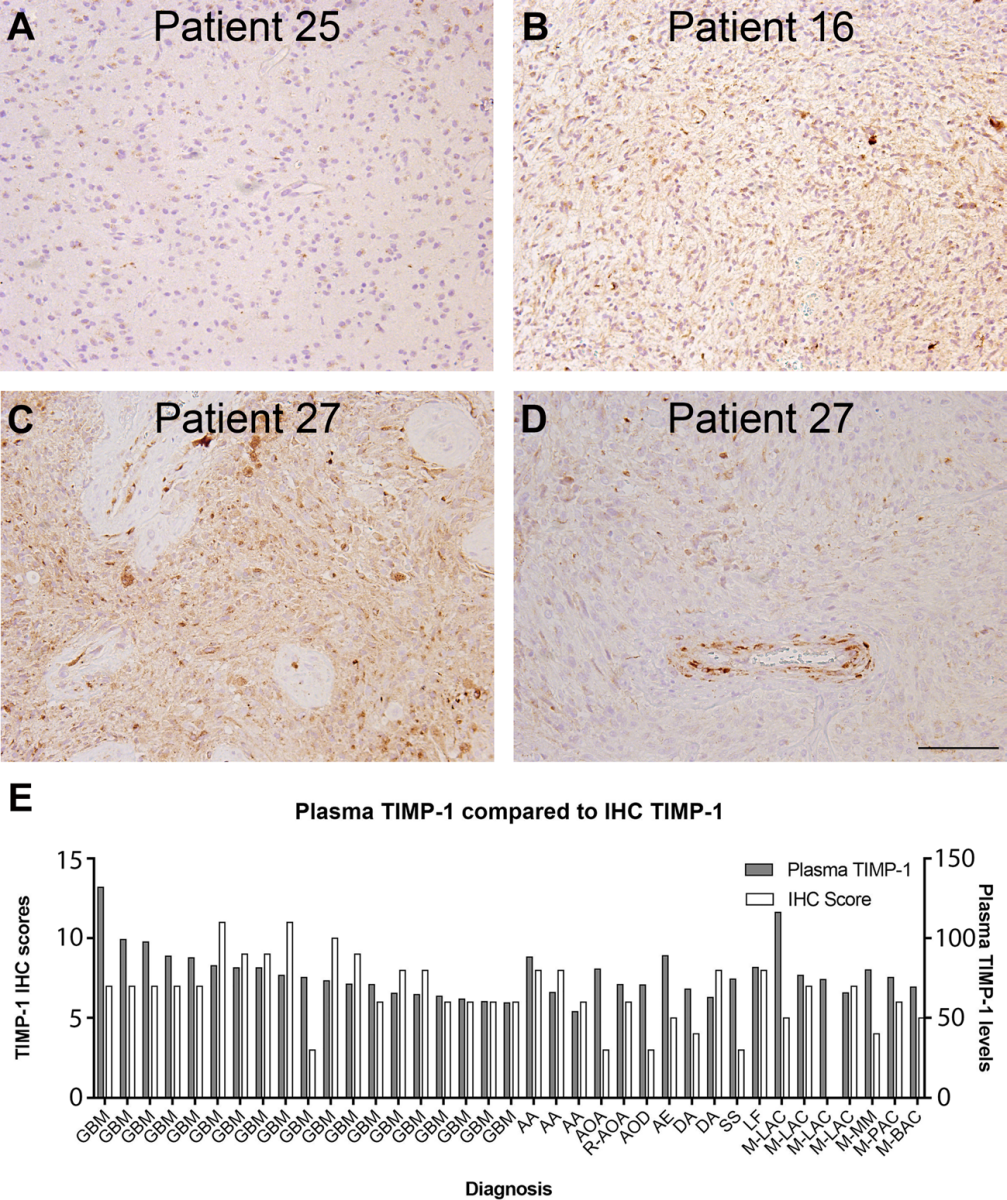
Plasma TIMP-1 was measured by an ELISA assay, which measures both free and complexed TIMP-1. The plasma TIMP-1 level was measured in 43 brain tumor patients and TIMP-1 immunohistochemistry was performed on 37 of the corresponding biopsies. No convincing variations were seen in plasma TIMP-1 levels however, variations were seen in the TIMP-1 protein expression in the biopsies (**a**–**d**). A high plasma level was not necessarily associated with a high TIMP-1 level in the biopsy (**a**) and high TIMP-1 levels were detected in several glioblastoma biopsies (**b, c**). The lowest plasma level was measured in patient 27 (**c, d**), but in the corresponding biopsy, areas with high levels of TIMP-1 positive tumor cells (**c**) as well as TIMP-1 positive tumor blood vessels (**d**) were detected. Distribution of plasma TIMP-1 levels and TIMP-1 immunohistochemical scores from 37 brain tumor patients is shown in (**e**). TIMP-1 was measured in blood samples prior to brain tumor surgery and shown as 10^−1^ ng TIMP-1/µg protein (**e**). When comparing the plasma TIMP-1levels and TIMP-1 immunohistochemical scores, no correlation was found. The diagnoses were: *DA* diffuse astrocytoma, *AA* anaplastic astrocytoma, *AOD* anaplastic oligoastrocytoma, *AOD* anaplastic oligodendroglioma, *GBM* glioblastoma, *R-AOA* recurrent anaplastic oligoastrocytoma, *AE* anaplastic ependynoma, *SS* synovial sarcoma, *LF* lymphoma, *M-LAC* metastasis from lung adenocarcinoma, *M-MM* metastasis from malignant melanoma, *M-PAC* metastasis from prostate adenocarcinoma, *M-BAC* metastasis from breast adenocarcinoma. *Scale bar* 100 µm

In addition, corresponding tumor biopsies were immunohistochemically stained for TIMP-1 and scored (Table 2; Fig. 3). The staining was performed on full slides and the scores given represented mean total scores for the whole vital tumor area. Six biopsies were excluded due to lack of viable tumor tissue, leaving 37 of the 43 for analysis. In general, no association was seen between TIMP-1 plasma levels and total TIMP-1 immunohistochemical scores (Fig. 3e). Furthermore no association was seen between TIMP-1 in plasma and TIMP-1 tumor cell score or TIMP-1 vessel score (data not shown). A high plasma level was not necessarily associated with a high TIMP-1 immunoreactivity in the biopsy (Fig. 3a) and high TIMP-1 immunoreactivity was detected in many glioblastoma biopsies (Fig. 3b) with normal plasma TIMP-1 (Table 2). The lowest plasma level was measured in patient 27 (Fig. 3c, d), but in the corresponding biopsy, areas with both high TIMP-1 tumor cell immunoreactivity (Fig. 3c) as well as areas with low TIMP-1 tumor cell immunoreactivity but presence of TIMP-1 positive tumor blood vessels (Fig. 3d) were detected illustrating the heterogenic nature of the TIMP-1 staining.

## Discussion

In the present study we performed *TIMP-1* FISH analysis on 33 glioblastomas (cohort II) to elucidate the frequency of *TIMP-1* gene aberrations. Furthermore, we measured TIMP-1 plasma levels in 43 brain tumor patients including 20 glioblastoma patients (cohort I). *TIMP-1*/CEN-X ratios and plasma levels were then compared to the immunohistochemical TIMP-1 expression in the corresponding biopsies.

FISH analysis is a powerful method for implementing biomarkers in the clinic. It is easy to perform on the paraffin embedded tumor biopsies being available in pathology departments. The FISH method allows assessment of specific gene aberrations and the FISH signals are relatively easy to quantify. For example, co-deletion of 1p19q has prognostic value in oligodendroglial tumors when assessed by FISH analysis [16, 17], whereas amplification of the human epidermal growth factor receptor 2 (*HER2*) oncogene has predictive value in breast cancer [18–20]. By performing *TIMP-1* FISH we aimed to elucidate (1) if the *TIMP-1* gene was deleted or amplified in glioblastomas and (2) if the increased TIMP-1 immunoreactivity found in glioblastomas could be associated with *TIMP-1* gene amplification.

In general, the *TIMP-1*/CEN-X ratio was close to 1, but varied between 0.7 and 1.09, however, we did not have pre-defined cut-off values for *TIMP-1* amplification or deletion, since, to our knowledge, *TIMP-1* FISH analysis in glioblastomas or other cancers has not been described before. In general, cut-off definitions have been discussed for 1p/19q co-deletions as well as for *HER2* amplification. For the 1p/19q co-deletions, ratios ranging from 0.15 to 0.7 have been used as definition for deletions [21–23], whereas a ratio less than 0.5 was suggested in the guidelines written by the SIOP Europe Neuroblastoma Pathology, Biology and Bone Marrow Group [24]. Regarding *HER2* amplification, a ratio ranging between 1.8 and 2.2 has been considered as gene amplification although a ratio of 1.5 has been suggested as a more biological founded value [25, 26]. Based on these studies, it is suggested that the *TIMP-1*/CEN-X ratios found in the present study were within a normal range. In contrast to the rather constant FISH ratio, we detected a broader variation in the TIMP-1 immunoreactivities in the glioblastomas. However, we did not find any association between the TIMP-1 protein expression and *TIMP-1*/CEN-X ratios. Based on these results, elevated TIMP-1 immunoreactivity in glioblastomas should be explained by other mechanisms than gene amplification.

In several TIMP-1 biomarker studies, the TIMP-1 protein levels have been measured in pre-operative blood samples and it has been demonstrated that plasma TIMP-1 in breast and colorectal cancer are significantly associated with tumor grade as well as patient survival [6, 8, 27–36]. Since gliomas can be difficult to reach surgically and vital tumor tissue is often sparse a blood sample is much easier to obtain. We investigated whether plasma TIMP-1 protein levels collected prior to surgery correlated with TIMP-1 tumor immunoreactivity. The ELISA used in the present study is a well-established and thoroughly validated assay [13]. The ELISA measures the total levels of TIMP-1 including both uncomplexed and complexed TIMP-1. The TIMP-1 levels detected in plasma from the brain tumor patients did not differ significantly when compared to the TIMP-1 levels in healthy subjects matched by age and gender. However, in another study, serum angiogenic profiles of glioblastoma patients were characterized using a protein array [30]. The authors found increased serum levels of TIMP-1 when comparing 36 glioblastoma samples to five control patients, hospitalized for elective spinal surgery, with no consideration of age and gender. A possible explanation for the different results may be the different methods and materials used in the studies and especially the control group used for comparison. In the present study, we compared each glioblastoma sample to two healthy subjects matched by gender and age, since studies have suggested especially age and gender to significantly affect plasma TIMP-1 levels [7, 13].

We detected a median plasma TIMP-1 level of 80.1 ng/ml (range 59.5–131.9 ng/ml) in the glioblastoma patients. The median TIMP-1 level as well as the narrow range is rather low when compared to studies describing plasma TIMP-1 levels in breast and colorectal cancer using the same method and laboratory. In a recent study, 4509 individuals, examined for colorectal cancer, had a median TIMP-1 level of 88.4 ng/ml (range 27.4–1166.0 ng/ml) [7]. Out of the 4509 individuals, 294 of the patients were diagnosed with colorectal cancer. The median level in the colorectal cancer patients increased from 98.5 ng/ml in stage I to 137.3 ng/ml in stage IV, with a significantly higher median TIMP-1 level found in stage IV patients compared to stage I–III. When TIMP-1 plasma levels were measured in breast cancer patients [37] a plasma median level of 81.5 ng/ml (range 41.9–174.9 ng/ml) was found. However, when measuring the median TIMP-1 in 46 patients with advanced disease in the same study, a significantly higher TIMP-1 level of 108.7 ng/ml (59.7–560.7 ng/ml) was detected [37]. Even for the highly malignant glioblastomas in the present study, we did not detect significantly higher levels (59.5–131.9 ng/ml) when compared to controls (65.0–84.3 ng/ml). The blood brain barrier may play a role in the lack of increased TIMP-1 protein in blood samples from glioblastoma patients. In the present study, however, we had access to the pre-operative MRI scans for the patients included. All glioblastomas showed contrast enhancement indicating interrupted blood-brain-barriers in these tumors.

In our previous study, the immunohistochemical expression of TIMP-1 in both tumor cells and blood vessels was investigated in 72 glioblastoma biopsies [3]. In 92 % of the tumors, TIMP-1 positive blood vessels were detected and in the present study TIMP-1 positive blood vessels were detected in 94.8 % of the biopsies and the possibility for the tumors to secrete TIMP-1 directly into the blood could therefore be present. However, based on the results obtained in the present study, the tumor TIMP-1 does not seem to reach the blood directly. In line with this, we were not able to find a clear correlation between TIMP-1 protein measured in pre-operative blood samples and TIMP-1 protein levels in the corresponding biopsies. This is in accordance with a previous study [37] where comparison of plasma TIMP-1 with TIMP-1 protein levels in tumor extracts from primary breast tumor patients, revealed a non-significant correlation.

The role of TIMP-1 as a biomarker has in recent years become increasingly interesting in relation to the second line treatment of glioblastomas consisting of the vascular endothelial growth factor antibody bevazicumab, administered in combination with the chemotherapeutic drug irinotecan [38–41]. In colorectal cancer it has been suggested that the TIMP-1 protein could be a possible new biomarker predicting the effect of treatment with irinotecan [42], and the potential of TIMP-1 as a predictive biomarker in glioblastomas needs to be further investigated in regard to sensitivity to irinotecan treatment.

In conclusion, the variations in TIMP-1 immunoreactivity observed in the biopsies could not be recognized in plasma TIMP-1 levels or be explained by *TIMP-1* gene aberrations. The present study suggests that scoring of TIMP-1 immunoreactivity is a better choice than measuring of TIMP-1 plasma levels or using *TIMP-1*/CEN-X ratios, in future studies evaluating TIMP-1 as a biomarker in glioblastomas.

## Electronic supplementary material

Below is the link to the electronic supplementary material.

Supplementary material 1 (PDF 51 KB)
