# Supplementary material for: Comparative studies of TIMP-1 immunohistochemistry, TIMP-1 FISH analysis and plasma TIMP-1 in glioblastoma patients
Source: J Neurooncol. 2016 Sep 12;130(3):439–48. doi: 10.1007/s11060-016-2252-4 (PMC5118392; doi:10.1007/s11060-016-2252-4)
Supplement: Supplementary file 1 — Supplementary material 1 (PDF 51 KB) [file 11060_2016_2252_MOESM1_ESM.pdf]

**Online Resource 1**

Comparative studies of TIMP-1 immunohistochemistry, TIMP-1 FISH analysis and plasma TIMP-1 in glioblastoma patients.

Journal of Neuro-Oncology

Charlotte Aaberg-Jessen[1,2], Bo Halle[1,3], Stine S. Jensen[1], Sven Müller[5], Unni Maria Rømer[4], Christian B. Pedersen[3], Nils Brünner[4*] and Bjarne W. Kristensen[1,6*]

[*]These authors contributed equally to the study

[1]Department of Pathology, Odense University Hospital, Denmark
[2]Department of Nuclear Medicine, Odense University Hospital, Denmark
[3]Department of Neurosurgery, Odense University Hospital, Denmark.
[4]Section of Molecular Disease Biology, Department of Veterinary Disease Biology, Faculty of Health and Medical Sciences, University of Copenhagen, Denmark
[5]DAKO, Glostrup, Denmark
[6]Institute of Clinical Research, University of Southern Denmark, Denmark

**Development of TIMP-1 probe mixture and FISH analysis**

The BAC clone (RP11-466C12) was identified by analysis of a 400 kb area around the TIMP-1 gene using the UCSC genome browser (http://genome.ucsc.edu). The BAC clone is covering the following previously identified genes: ARAF wild-type allele (ARAF), human synapsin I (SYN1), tissue inhibitor of metalloproteinases-1 (TIMP-1), complement factor properdin (CFP), ELK1, ubiquitously expressed transcript (UXT), and AK094108. The BAC clone was cultured in LB medium (Sigma Aldrich) supplemented with 12.5 µg/mL chloramphenicol (Sigma Aldrich) and purified according to the alkaline purification of BAC (Poulsen, TS, Methods in Molecular Biology, 2004). The clone was verified using in silico BamHI digest of the DNA sequence from the UCSC and compared with a BamHI endonuclease digestion of the purified BAC clone as recommended by the enzyme manufacturer (Invitrogen).

The TIMP-1 probe mixture was developed by Dako A/S, Denmark. The probe BAC DNA was labeled by nick translation with Texas Red-5-dCTP (Millipore) as described by the manufacturer (Roche Diagnostics). A total of 10 ng/µL labeled DNA was used for FISH and suppression of undesired background staining derived from repetitive sequences was achieved using specific PNA Alu sequence oligos (Nielsen *et al*., Nova Science Publishers, 2006). A fluorescein labeled mixture of PNAs specific for the chromosome X □- satellite sequences (CEN-X PNA probe) was used as a reference for the copy number of chromosome X. The PNAs were supplied by Dako Denmark A/S, Denmark . Figure 1 shows a schematic representation of chromosome X and the localization of the part of region Xp11 covered by the BAC DNA as well as the area of centromere X covered by the CEN-X PNA probe.

FISH analysis was performed on two TMAs described above, in order to elucidate TIMP-1 gene copy number. The FISH procedure was performed according to Dako Histology FISH Accessory Kit (K5599). In brief, paraffin sections were deparaffinized and rehydrated. The sections were placed in pre-treatment buffer and pre-treatment was performed using a microwave oven. Subsequently, the sections were incubated with RTU-pepsin for 2 min at 37ºC and washed twice for 3 min. Slides were air-dried for 15-20 min, followed by application of 10 µl probe mix. Hybridization was carried out for approximately 20 h using a melting temperature of 82ºC and a hybridization temperature of 45ºC. Coverslips were mounted using fluorescence mounting medium counter staining the cell nuclei.
